# Utero-Gestation Table

**Published:** 1878-02-20

**Authors:** 


*TABLE FOR CALCULATING THE PERIOD OF UTERO-GESTATION.*

| NINE CALENDAR MONTHS. | | DAYS | 10 LUN. M'THS | DAYS |
|---|---|---|---|---|
| From | To | | To | |
| January ..1 | Septemer 30 | 273 | October....7 | 280 |
| February. 1 | October.. 31 | 273 | November..7 | 280 |
| March ... 1 | Nov..... 30 | 275 | December ..5 | 280 |
| April.....1 | Dec..... 31 | 275 | January... 5 | 280 |
| May ....e 1 | January 31 | 276 | February,..4 | 280 |
| June .....1 | February 28 | 273 | March.....7 | 280 |
| July .....1 | March.. 31 | 274 | Apr ...... 6 | 280 |
| August ...1 | April....30 | 273 | May ...... 7 | 280 |
| September 1 | May.... 31 | 273 | June...... 7 | 280 |
| October ..1 | June.... 30 | 273 | July...... 7 | 280 |
| November 1 | July.... 31 | 273 | August.... 7 | 280 |
| December 1 | August... 31 | 274 | September..6 | 280 |

The above obstetric "Ready Reckoner" consists of two columns, one of Calender the other of Luna months, and may be read as follows: A patient has ceased to menstruate on the 1st of July; her confinement may be expectod at soonest about the 31st of March (the end of nine calendar months); or at latest, on the 6th of April (the end of ten Luna months). Another has ceased to menstruate on the 20th of January; her confinement may be expected on the 30th of September, plus 20 days (the end of nine calendar months) at soonest; or on the 7th of October (the end of ten Luna months), at latest.—*Lindsay & Blakiston's VisitingList.*

FOR ENLARGED LIVER.

Use the nitro muriatic bath as described by Eberle, or may find it in U. S. Dispensatory, and give internally, Iodide of Potassium and Ext. of Taraxicum.

A GOOD LIVER PILL:

R—Podophyllin........................gr. iv
Capsicum..............................gr. iv
Pulv. Rhei............................gr. xv

For twelve pills. One every other night.

